# Gene signature related to cancer stem cells and fibroblasts of stem‐like gastric cancer predicts immunotherapy response

**DOI:** 10.1002/ctm2.1347

**Published:** 2023-07-30

**Authors:** Ji‐Yong Sung, Jae‐Ho Cheong

**Affiliations:** ^1^ Center for Genome Engineering Institute for Basic Science Daejeon Republic of Korea; ^2^ Department of Surgery Yonsei University College of Medicine Seoul Republic of Korea

Dear Editor

The stem‐like patient group does not respond to immunotherapy and standard treatment and has the worst prognosis.^1^, ^2^ We attempted to uncover signatures associated with stem‐like types from multiple viewpoints and predict immunotherapy responses utilizing stem‐like signature genes discovered at the single‐cell level. We demonstrated that the stem‐like signature from PAM965 (Table S1) was enriched in cancer stem cells (CSCs) and fibroblasts in two distinct gastric cancer (GC) single‐cell cohorts (Figure 1A–C). In cohort 1, cells with exceptionally high stem‐like signature scores were found among tumour cells. Highly stem‐like tumour cells exhibited cancer hallmarks and CSC characteristics (Figure 1D, Figure S1A,B). In cohort 2, the stem‐like signature was enriched in fibroblasts (Figure 1C).^3^ We confirmed that 14 pathways were highly upregulated in stem‐like tumour cells (Figure 1D). Furthermore, we identified differentially expressed genes (DEGs) by distinguishing tumour cells with highly stem‐like signatures from others, as well as the most significantly enriched pathways: blood vessel development and NABA core matrix signalling (Figure 1E).^4^ SIG276 is genes in the cells that exhibited CSC characteristics and differed from other tumour cells were defined as signature 276. We identified 276 CSC markers (Table S2) of the stem‐like type at the single‐cell level, which were otherwise difficult to identify in the bulk stem‐like type patient group.

**FIGURE 1:**
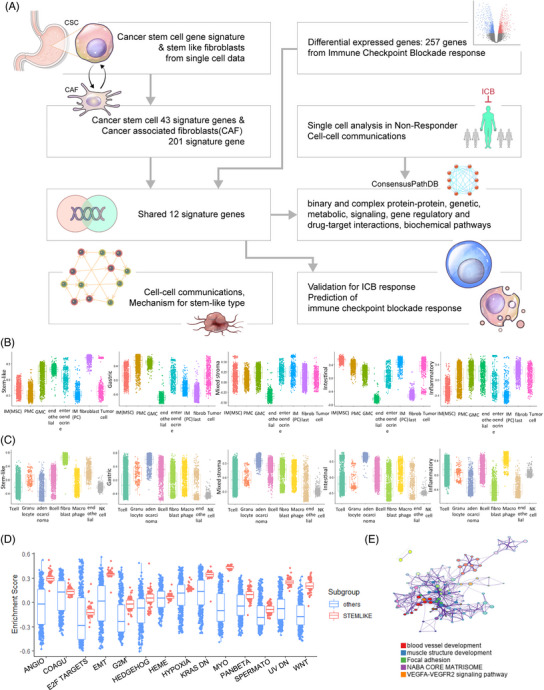
Stem‐like signature genes are enriched in fibroblasts and cancer stem cells at the single‐cell level. (A) Analysis pipeline for this study. (B) Box plot of the eight single‐cell type (including immune cells) enrichment scores for the five molecular signatures in gastric cancer. (C) Box plot of the eight cell‐type enrichment scores for the five molecular signatures at the single‐cell level in gastric cancer. (D) Box plot of the 14 cancer hallmarks of stem‐like tumour cells highly expressed at the single‐cell level. (E) Gene ontology network for differentially expressed genes between stem‐like tumour cells and others at the single‐cell level.

Based on five molecular subtypes, we identified 276 up‐regulated genes between stem‐like and other tumour cells at the single‐cell level in the bulk Y497 cohort (Figure 2A). SIG276 activity was significantly high in the stem‐like type (Figure 2A). Kaplan–Meier survival analysis of TCGA‐STAD data revealed poor prognosis in the group with increased SIG276 expression (*p* = .022) (Figure 2B). Moreover, we identified 110 genes with significantly high expression in stem‐like tumours through DEG analysis of stem‐like tumour cells and fibroblasts at the single‐cell level. Using protein–protein interaction (PPI) analysis,^5^ most of these genes were enriched in the Rho GTPase signalling pathway (Figure 2C). Using TCGA‐STAD data, a poor prognosis was confirmed in the group with a high expression of 108 signature genes (Figure S1C). We found stem‐like tumour cells and other types of tumour cells (mixed stroma, gastric, inflammatory, intestinal), except for common genes among the 276 signatures and 110 genes (Table S3) highly expressed in stem‐like tumour cells that differed between stem‐like tumour cells and fibroblasts. Forty‐three genes (SIG43) (Table S4) were highly expressed in the stem‐like type of the five molecular subtypes of Y497 bulk samples (Figure 2D). We also performed a Kaplan–Meier survival analysis on TCGA‐STAD data using SIG43 (Figure S2E). Their PPIs were primarily enriched in the nuclear factor of activated T‐cells transcription factor (NFAT TF), CD8 T‐cell receptor (TCR) downstream, and AP1 pathways (Figure 2F). We analyzed ConsensusPathDB (CPDB) using 276 genes and identified EGR1 and JUN as hub proteins in the PPI network (Figure 2G). These genes were regulated by transcription factors, such as RBMX, SRF, and ESR1 (Figure 2H). We used SIG43 to determine patient prognosis in the Y497 cohort and found poor prognosis in the high‐expression group (*p* < .001) (Figure 2I). Lapatinib, *S*‐trityl‐l‐cysteine and GW843682X were highly sensitive drugs targeting *JUN*, whereas rapamycin targeted *EGR1* (Figure 2J).

**FIGURE 2:**
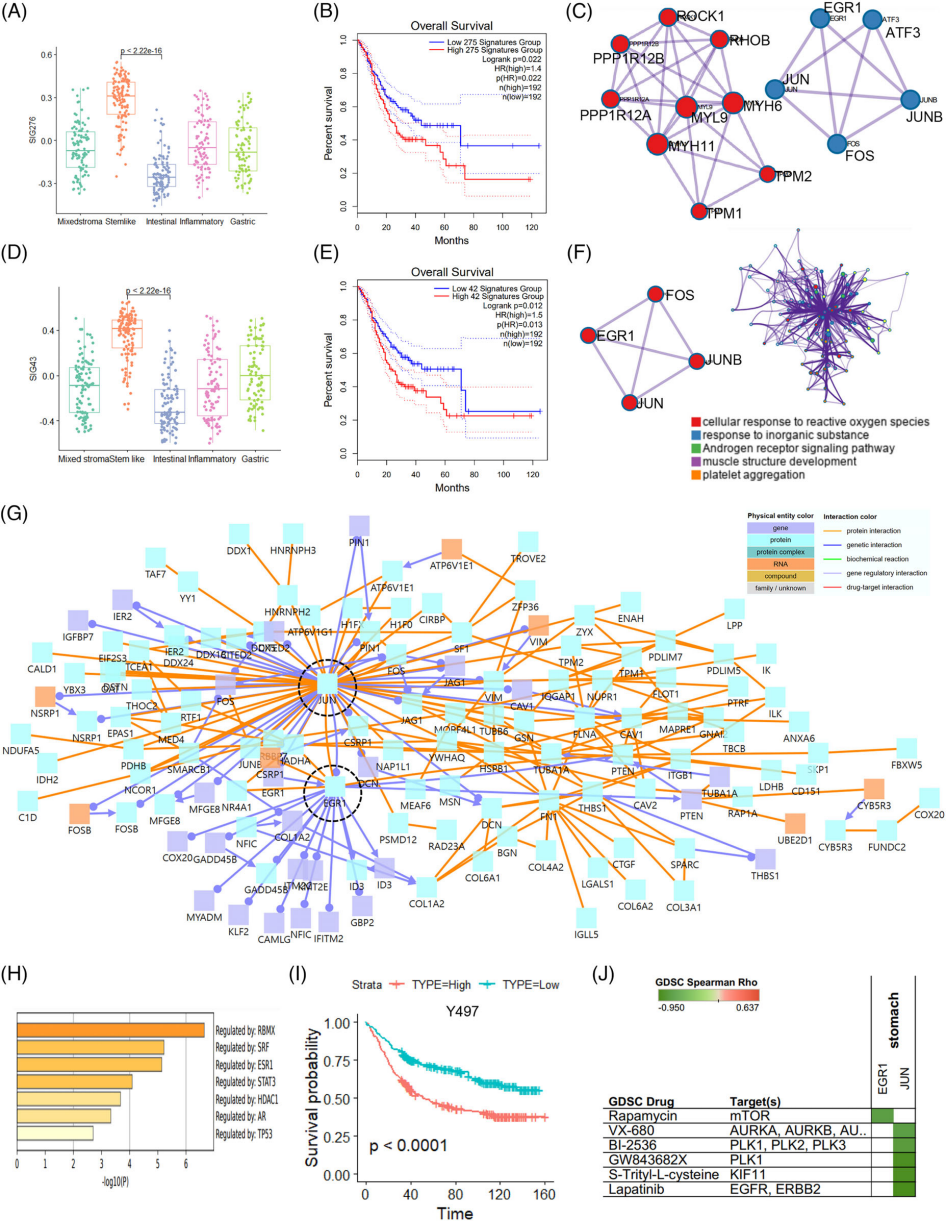
The stem‐like tumour cell signature is enriched in stem‐like bulk samples. (A) Box plot of the 276 signature genes (SIG276) in the Y497 hospital cohort. (B) Kaplan–Meier plots showing the overall survival rates for high‐ and low‐SIG276 activity samples in The Cancer Genome Atlas (TCGA) data related to stomach adenocarcinoma. (C) Protein–protein interaction (PPI) network of the 110 differentially expressed genes between tumour cells and fibroblasts. (D) Box plot of the 43 signature genes (SIG43: only stem‐like tumour cell‐specific up‐regulated genes) in the Y497 hospital cohort. (E) Kaplan–Meier plots showing the overall survival rates of the high‐ and low‐SIG43 activity samples in TCGA data related to stomach adenocarcinoma. (F) Enriched biological PPI network and gene ontology analysis for SIG43. (G) PPI network for SIG43. (H) Bar graph of the transcriptomic factors for SIG43. (I) Kaplan–Meier plots showing the overall survival rates for SIG43‐high and ‐low samples in the Y497 hospital cohort. (J) Drug and target gene prediction for stomach cancer cells using genomics of drug sensitivity in cancer (GDSC). The green colour (negative correlation) shows high drug sensitivity.

By analyzing the DEGs in 85 CSCs and fibroblasts, we confirmed that 455 genes were up‐regulated only in fibroblasts (Figure 3A). StemID analysis identified 502 genes highly expressed in high‐stemness fibroblasts (Figure 3B). In total, 201 highly expressed genes (SIG201) in high‐stemness fibroblasts were enriched in Rho GTPase signalling (Figure 3C). We identified SIG201 as a cancer‐associated fibroblast signature with high entropy and validated its differential enrichment depending on the stemness of the fibroblasts in the cluster (Figure 3D,E). These 201 markers were significantly expressed in fibroblasts, but nearly non‐existent in CSCs (Figure 3F). Using TCGA‐STAD dataset, we identified poor prognosis associated with high expression of SIG201 (*p* = .00081) (Figure 3G). We established that SIG201 enriched the stem‐like type in the five molecular subtype datasets of 497 patients with GC at Yonsei Hospital (Figure 3H). Additionally, we validated SIG43 and SIG201, which we discovered in 88 different samples (primary tumour, patient‐derived organoid, patient‐derived xenograft and patient‐derived xenograft organoid) (Figure 3I–K) (Supplementary Method).

**FIGURE 3:**
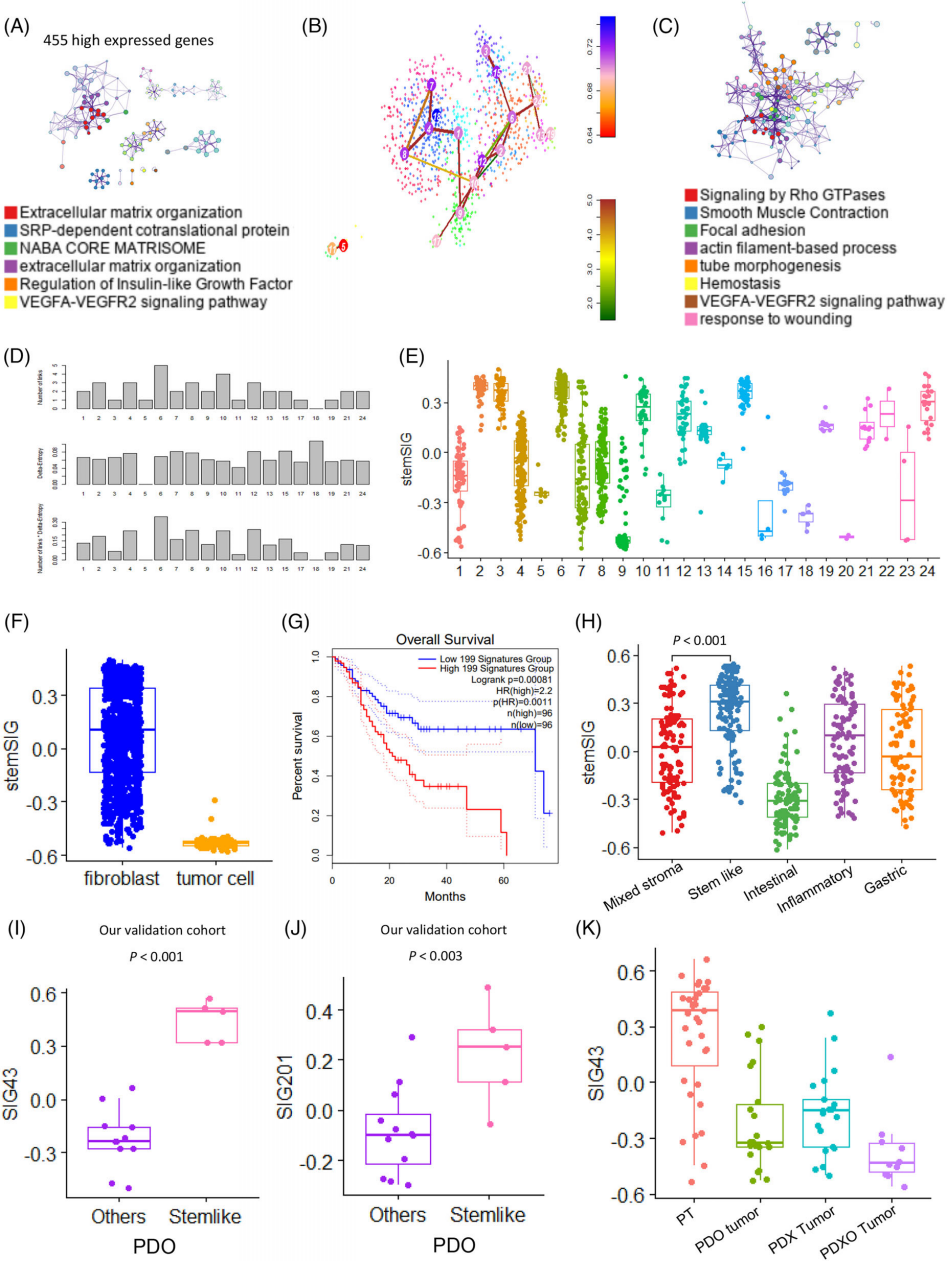
The 201 activated fibroblast signature genes (SIG201) is enriched in stem‐like type and indicate poor patient prognosis. (A) Highly differentially expressed gene networks for fibroblasts. (B) T‐distributed stochastic neighbor embedding plot for fibroblasts and cancer stem cells. (C) Highly enriched biological pathways in high‐stemness fibroblasts. (D) Bar graph of entropy in the stem clusters. (E) Box plot of SIG201 for fibroblast scores in stem clusters. (F) Box plot of SIG201 fibroblast scores in fibroblasts and tumour cells. (G) Kaplan–Meier plots showing the overall survival rates for the high‐ and low‐SIG201 groups. (H) Box plot of SIG201 expression in the five molecular subtypes. (I) Box plot of the 43 signature genes (SIG43) in patient‐derived organoid (PDO) types. (J) Box plot of SIG201 in PDO types. (K) Box plot of SIG43 in the four different sample types.

We investigated 257 (Table S5) distinct genes in patients with immune checkpoint blockade (ICB) responses to GC.^6^, ^7^ In the non‐responder group,^6^, ^8^ 257 genes were up‐regulated (Figure 4A). In this study, 12 common genes (SIG12) (Table S6) were discovered among SIG43 for CSCs, SIG201 for fibroblasts, and 257 ICB‐related non‐response genes. We confirmed that the stromal score was significantly higher in the non‐responder group for ICB response (Figure 4B), and genes in the non‐responder group were enriched in the *CAMKK2* pathway (Figure 4C). We examined outgoing and incoming signalling patterns at the single‐cell level (Figure 4D) to investigate how cell–cell interactions differed based on the cell types in non‐responder patients. The incoming fibroblast signalling patterns revealed the high relative intensity of the ligands of midkine (MK), pleiotrophin (PTN), PERIOSTIN, fibroblast growth factors (FGF)^9^ and growth arrest specific (GAS) (Figure 4E). We demonstrated poor prognosis (*p* = 8.0 × 10^−4^) at high SIG12 expression in TCGA‐STAD dataset (Figure 4F) and compared their expression based on the ICB response of patients with GC at Samsung Medical Center.^6^ Ten genes were expressed considerably higher in the non‐responder group (Figure 4G). We used CPDB^10^ to examine binary and complex PPIs, and *MMP2*, *FBLN1* and *VCAN* ^7^ served as hub genes (Figure 4H). SIG12 were enriched in the stem‐like type among the five molecular subtypes (Figure 4I). We examined SIG12 expression in ICB responders as a validation cohort and found a considerably higher expression in the non‐responder group (Figure 4J). Finally, 12 signature genes associated with the non‐responder group were substantially expressed as ICB response biomarkers. These findings helped us comprehend the specific processes in stem‐like cell types and utilize SIG12 to anticipate the ICB response. Our novel findings propose a therapeutic strategy targeting the aggressive and recalcitrant stem‐like tumour cells of GC.

**FIGURE 4:**
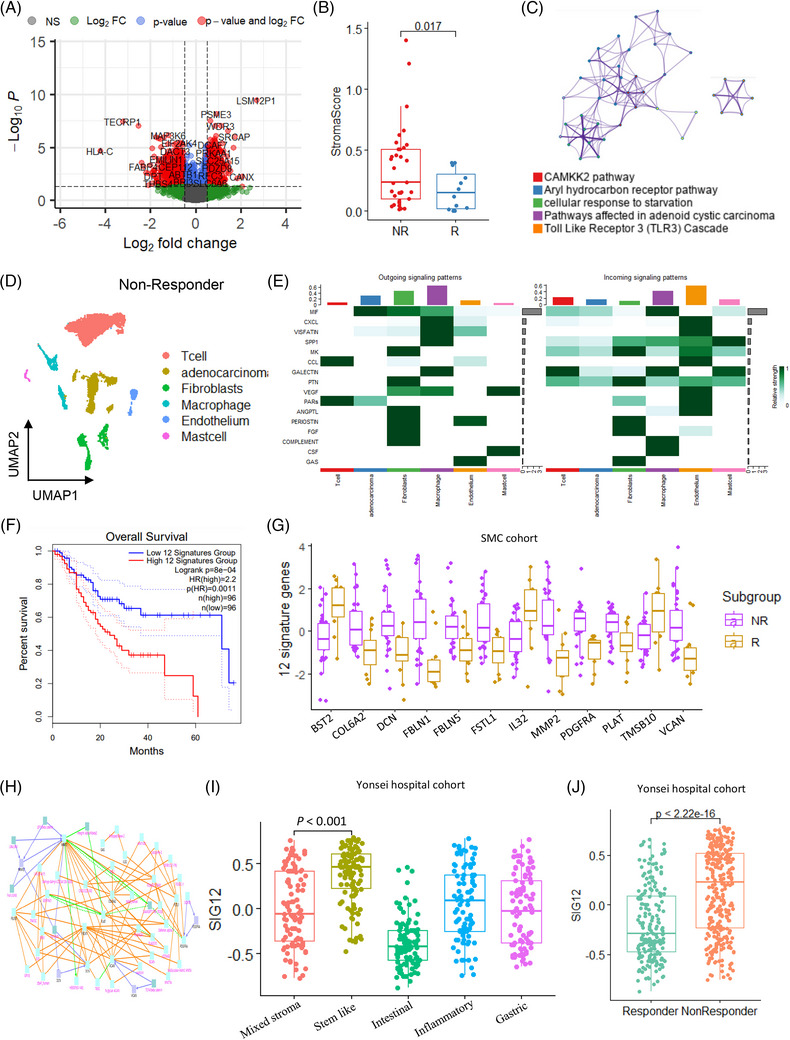
Twelve signature genes (SIG12) predict immune checkpoint blockade response in gastric cancer. (A) Volcano plot of differentially expressed genes in the responder and non‐responder groups. (B) Box plot of the stromal score in non‐responder and responder groups. (C) Enriched biological pathways of up‐regulated genes. (D) Uniform manifold approximation and projection of the six cell types in non‐responders. (E) Heat map of outgoing and incoming signalling patterns in the non‐responder group. (F) Kaplan–Meier plots showing the overall survival rates for the high‐ and low‐SIG12 groups. (G) Box plot for SIG12 expression in non‐responder and responder groups. (H) Protein–protein interaction network. (I) Box plot for SIG12 in the five molecular subtypes. (J) Box plot for SIG12 in responder and non‐responder groups.

## FUNDING INFORMATION

The National R&D Program for Cancer Control, Ministry of Health and Welfare, Republic of Korea, Grant Number: HA22C0050

## CONFLICT OF INTEREST STATEMENT

The authors declare that they have no competing interests.

## Supporting information

Supplementary Information

Supplementary Information

Supplementary Information
