# Supplementary figures and images for: Gene signature related to cancer stem cells and fibroblasts of stem‐like gastric cancer predicts immunotherapy response
Source: Clin Transl Med. 2023 Jul 30;13(8):e1347. doi: 10.1002/ctm2.1347 (PMC10387327; doi:10.1002/ctm2.1347)

## Slide 1
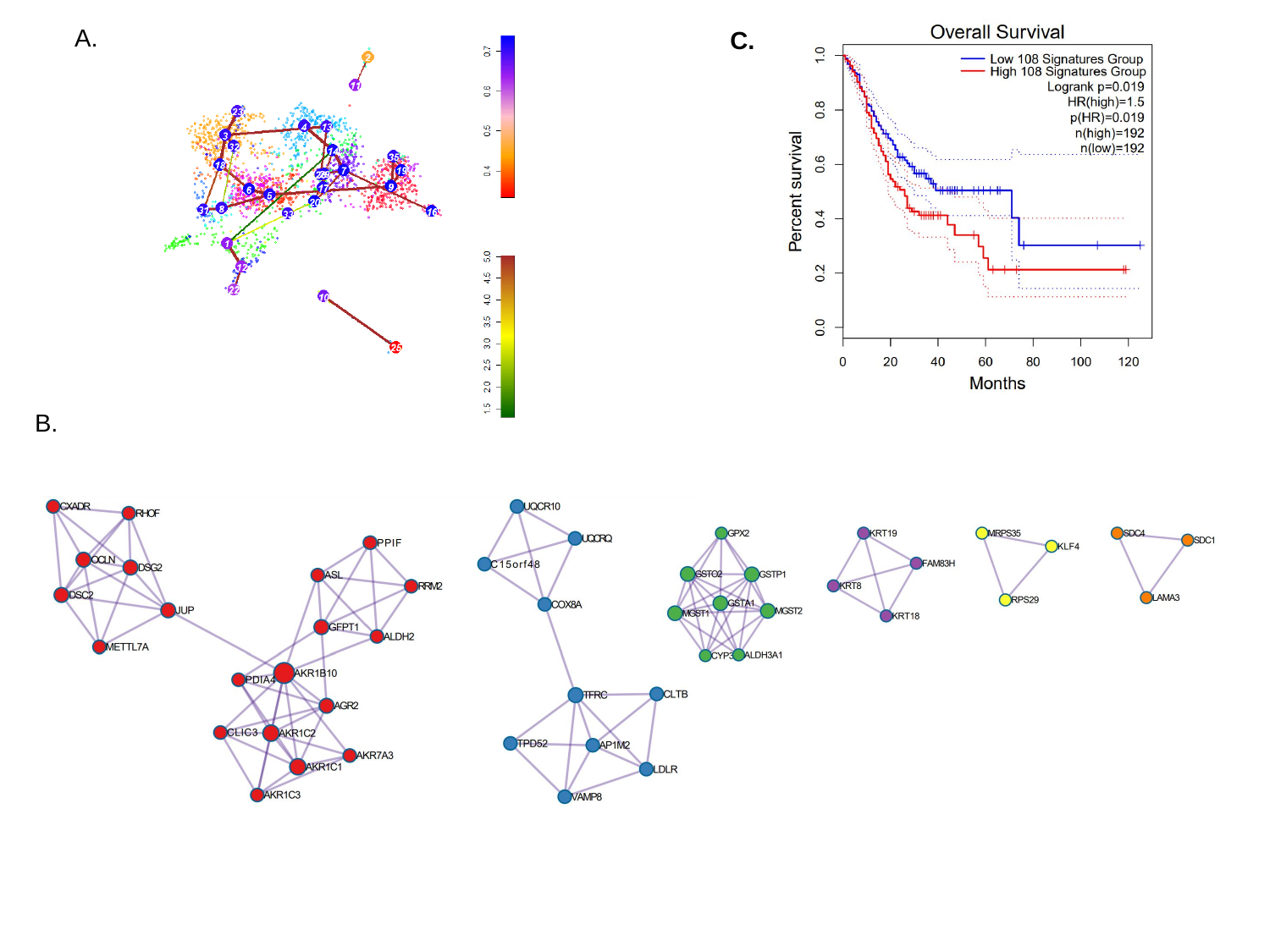

A.
C.
B.

Supplement: Supplementary file 1 — Supplementary Information [file CTM2-13-e1347-s002.pptx]
