# Supplementary material for: Gene signature related to cancer stem cells and fibroblasts of stem‐like gastric cancer predicts immunotherapy response
Source: Clin Transl Med. 2023 Jul 30;13(8):e1347. doi: 10.1002/ctm2.1347 (PMC10387327; doi:10.1002/ctm2.1347)
Supplement: Supplementary file 3 — Supplementary Information [file CTM2-13-e1347-s003.docx]

**Methods**

**Generation and culture of normal gastric and tumor organoids**

Clinical samples used for organoid establishment were obtained from patients who had provided informed consent at Yonsei University Cancer Hospital after the approval of the institutional review board (IRB No. 4-2017-0106). Healthy and cancer specimens were collected by surgical resection, endoscopic biopsy, or ascites drainage. Organoids were generated and maintained as previously described (1) with minor modifications. Surgical specimens were vigorously washed with PBS and minced into ~3 mm3 fragments using surgical scissors. The tumor fragments were further enzymatically digested with 1.5% collagenase at 37 °C for 60 min. Meanwhile, the normal fragments were treated with 10 mM EDTA at room temperature for 10 min.

Before plating, collected epithelia were washed with phosphate buffered saline (PBS) supplemented with 10% fetal bovine serum (FBS) to inactivate digestive enzymes. Ascites samples were centrifuged and washed three times with ice-cold PBS, and sedimented cells were used for organoid culture. Isolated gastric cells were embedded in Matrigel droplets and overlaid with culture medium. Culture conditions were as follows: Advanced Dulbecco’s modified Eagle medium/F12 medium (Invitrogen, Carlsbad, CA, USA), R-spondin-conditioned medium, and Wnt-conditioned medium supplemented with gastric growth factors including bone morphogenetic protein inhibitor, noggin (PeproTech, Cranbury, NJ, USA), GlutaMAX-I (Invitrogen), B27 (Invitrogen), TGF beta I A83-01 (TOCRIS, Minneapolis, MN, USA), ROCK inhibitor, nicotinamide (Sigma-Aldrich, St. Louis, MO, USA), N-acetylcysteine (Sigma-Aldrich), gastrin (Sigma-Aldrich), epidermal growth factor (PeproTech), and fibroblast growth factor 10 (R&D systems, Minneapolis, MN, USA). The media were changed every 3 or 4 days. Gastric organoids were subsequently passaged every 6 to 8 days, depending on the size of the organoids. Organoid size was measured as an average of the shortest and longest diameters crossing the center of the organoids [1].

**Transcriptomic analysis of the five molecular subtypes**

The molecular subtypes of GC are classified according to the following characteristics: 1) immune subtype, defined by the positive expression of both granzyme B (GZMB) and tryptophan tRNA synthetase (WARS); 2) epithelial subtype, defined by the positive expression of caudal type homeobox 1 (CDX1); 3) stem-like subtype, defined by the positive expression of the secreted frizzled-related protein (SFRP4) [1]. Clinically, the immune subtype is associated with the most favorable prognosis but is not responsive to chemotherapy. The epithelial subtype, which is sensitive to chemotherapy, has an intermediate prognosis [1]. The stem-like subtype is typically associated with the worst prognosis and is not responsive to standard chemotherapy or immune-directed therapy [1].

We used microarray data in our previous study [2] to classify the five molecular subtypes (stem-like, mixed stroma, intestinal, inflammatory, and gastric) with the non-negative matrix factorization algorithm and defined prediction analysis of microarrays (PAM965) for each molecular subtype. PAM is a statistical method for classifying gene expression data, as demonstrated by studies using nearest shrunken centroids [3].

Four GC cohorts (TCGA-STAD, Y497, GSE62254, and GSE15459) were used for transcript analysis. Kaplan–Meier survival plots were generated using the “survival” package in R. To analyze the 50 cancer hallmark pathways, a gene set was downloaded from MSigDB (http://www.gsea-msigdb.org/gsea/msigdb/genesets.jsp?collection=H), and pathway activity was analyzed using the Gene Set Variation Analysis (*GSVA*) R package [4]. The signature gene for each molecular subtype was obtained from PAM965 using the non-negative matrix factorization (NMF) algorithm described in a previous study. Metascape [5] was used for the Gene Ontology (GO) analysis of signature genes in each molecular subtype. For deconvolution analysis, the characteristics of immune cells were analyzed using CIBERSORT [6]. Using TCGA-STAD data, a poor prognosis was confirmed in the group with a high expression of 108 signature genes (**Supplementary Figure 1C**). The immune checkpoint blockade response of the Y497 cohort was predicted by TIDE [7].

**Single-cell RNA-seq analysis of the five molecular subtypes**

We downloaded single-cell RNA-seq data (https://dna-discovery.stanford.edu/research/datasets/) from a previous study on single-cell RNA-seq analysis [8, 9]. MAGIC imputation was performed for the meta-analysis, and cell types were classified using marker genes from previously published studies [10]. The activity levels of the five molecular subtype signatures were analyzed using the GSVA algorithm [4], and accuracy was improved by conducting more than 1,000,000 runs. The same gene set used in the bulk sample was used as cancer hallmarks across cell types. To calculate the stemness of a single cell, the StemID tool was used [11]. The *DEsingle* R package [12] was utilized for identifying single-cell DEGs for each cell type. Metascape was used for protein–protein interaction (PPI), transcriptional factor (TF), and GO analyses. All statistical criteria required FDR < 0.001. For cell–cell communication analysis at the single-cell level, CellChat was used [13].

We first confirmed the activation of stemness, according to the entropy of stem-like type cells, most of the cells had very high stemness and entropy (**Supplementary Figure 1A**). We then divided the cells into five categories based on the protein–protein interaction (PPI) of highly regulated genes. Among them, drug metabolism was enriched in cytochrome P450 and glutathione metabolism (**Supplementary Figure 1B**).

**Other analyses**

For the TCGA-STAD survival analysis, GEPIA2 was used [14]. The PPI network was analyzed through the ConsensusPathDB-human (CPDB) (<http://cpdb.molgen.mpg.de/>) network [15], and hub proteins were identified. Target genes and drugs were predicted using Genomics of Drug Sensitivity in Cancer(GDSC) [16].

1. Bartfeld, S., et al., *In vitro expansion of human gastric epithelial stem cells and their responses to bacterial infection.* Gastroenterology, 2015. **148**(1): p. 126-136 e6.

2. Cheong, J.H., et al., *Predictive test for chemotherapy response in resectable gastric cancer: a multi-cohort, retrospective analysis.* Lancet Oncol, 2018. **19**(5): p. 629-638.

3. Tibshirani, R., et al., *Diagnosis of multiple cancer types by shrunken centroids of gene expression.* Proc Natl Acad Sci U S A, 2002. **99**(10): p. 6567-72.

4. Hanzelmann, S., R. Castelo, and J. Guinney, *GSVA: gene set variation analysis for microarray and RNA-seq data.* BMC Bioinformatics, 2013. **14**: p. 7.

5. Zhou, Y., et al., *Metascape provides a biologist-oriented resource for the analysis of systems-level datasets.* Nat Commun, 2019. **10**(1): p. 1523.

6. Newman, A.M., et al., *Determining cell type abundance and expression from bulk tissues with digital cytometry.* Nat Biotechnol, 2019. **37**(7): p. 773-782.

7. Jiang, P., et al., *Signatures of T cell dysfunction and exclusion predict cancer immunotherapy response.* Nat Med, 2018. **24**(10): p. 1550-1558.

8. Sathe, A., et al., *Single-Cell Genomic Characterization Reveals the Cellular Reprogramming of the Gastric Tumor Microenvironment.* Clin Cancer Res, 2020. **26**(11): p. 2640-2653.

9. Kim, J., et al., *Single-cell analysis of gastric pre-cancerous and cancer lesions reveals cell lineage diversity and intratumoral heterogeneity.* NPJ Precis Oncol, 2022. **6**(1): p. 9.

10. van Dijk, D., et al., *Recovering Gene Interactions from Single-Cell Data Using Data Diffusion.* Cell, 2018. **174**(3): p. 716-729 e27.

11. Grun, D., et al., *De Novo Prediction of Stem Cell Identity using Single-Cell Transcriptome Data.* Cell Stem Cell, 2016. **19**(2): p. 266-277.

12. Miao, Z., et al., *DEsingle for detecting three types of differential expression in single-cell RNA-seq data.* Bioinformatics, 2018. **34**(18): p. 3223-3224.

13. Jin, S., et al., *Inference and analysis of cell-cell communication using CellChat.* Nat Commun, 2021. **12**(1): p. 1088.

14. Tang, Z., et al., *GEPIA2: an enhanced web server for large-scale expression profiling and interactive analysis.* Nucleic Acids Res, 2019. **47**(W1): p. W556-W560.

15. Herwig, R., et al., *Analyzing and interpreting genome data at the network level with ConsensusPathDB.* Nat Protoc, 2016. **11**(10): p. 1889-907.

16. Qin, Y., et al., *A tool for discovering drug sensitivity and gene expression associations in cancer cells.* PLoS One, 2017. **12**(4): p. e0176763.
